# Safety and efficacy of remifentanil target-controlled infusion for conscious sedation in a pregnant woman: a case report

**DOI:** 10.1186/s13256-023-04303-3

**Published:** 2024-03-07

**Authors:** Camilla Munafo’, Antonella Loperfido, Fulvio Mammarella, Arianna Crosti, Federico Iannilli, Francesca Romana Millarelli, Gianluca Bellocchi, Luigi Tritapepe

**Affiliations:** 1 Anaesthesia and Intensive Care Division, San Camillo-Forlanini Hospital Rome, Rome, Italy; 2 Otolaryngology Unit, San-Camillo Forlanini Hospital, Rome, Italy; 3 Anaesthesia and Intensive Care Division, University of Rome “La Sapienza”, Rome, Italy; 4 Anaesthesia and Intensive Care Division, University of Rome “Tor Vergata”, Rome, Italy

**Keywords:** Conscious sedation, Analgosedation, Remifentanil, Pregnancy, Functional endoscopic sinus surgery, FESS

## Abstract

**Background:**

Every year 2% of pregnant women undergo nonobstetric surgical interventions worldwide. According to the American College of Obstetricians and Gynecologists Committee on Obstetric Practice, pregnant women should never be denied the most appropriate surgical treatment, regardless of the trimester of pregnancy. However, additional attention should be paid during the first trimester since it has the highest risk of inducing teratogenic mutations; additionally, during the third trimester, due to the possibility of preterm birth and low birth weight of the newborn, great care should be paid.

**Case presentation:**

We present the case of a Caucasian 36-year-old woman during her 21st week of pregnancy, with a normal-sized fetus, according to the gestational age on ultrasound exam, and with no additional risk factors. The patient referred to an increasing nasal obstruction associated with rhinorrhea of the left nasal cavity. She also reported episodes of sleep apnea and hyposmia. The patient received a detailed otolaryngological examination, which allowed for identification of a mass within the left nasal cavity. The subsequent nasal endoscopy confirmed a grayish polypoid mass lesion with a multinodular surface occupying the entire left nasal fossa. The lesion totally obliterated the left maxillary sinus, resulting in obstruction of the anterior osteomeatal unit and ethmoidal sinusitis. She was referred for a functional endoscopic sinus surgery using analgosedation with remifentanil target-controlled infusion.

**Discussion and conclusion:**

To the very best of our knowledge, this is the first case described in English literature about the use of analgosedation with remifentanil target-controlled infusion for otolaryngology surgery, specifically in functional endoscopic sinus surgery. It could be an interesting option to avoid the use of inhaled anesthetics that could induce fetal damage, especially during the first months of pregnancy. Furthermore, patient intubation is not necessary, which avoids cases of difficult intubation or any trauma to the airways. An adequate informed consent and appropriate compliance are elements of paramount importance in tailoring the anesthetic strategy for pregnant women who need nonobstetric surgical management.

## Background

Every year, 2% of pregnant women undergo nonobstetric surgical interventions worldwide [1, 2].

According to the American College of Obstetricians and Gynecologists Committee on Obstetric Practice, pregnant women should never be denied the most appropriate surgical treatment, regardless of the trimester of pregnancy [3].

However, additional attention should be paid during the first trimester since there is the highest risk of inducing teratogenic mutations; similarly, during the third trimester, due to the possibility of preterm birth and low birth weight of the newborn, great care should be taken [4].

It is well known that when a pregnant woman receives drugs some of them cross the placenta, reaching fetal circulation through various mechanisms and possibly interacting directly with the fetus.

For instance, there are several studies performed in rats that have highlighted embryonic brain development after exposure to sevoflurane; additionally, propofol and desflurane may also induce neuronal apoptosis, resulting in cognitive dysfunction by inducing IL-6 production [5, 6].

In contrast, the use of opioids during pregnancy, especially remifentanil, is safer. This is mainly due to its peculiar pharmacokinetics; in fact, it has a fast onset and offset. Therefore, it does not interfere with the physiological changes of pregnancy, and despite passing the placenta, it is quickly metabolized and prevents the need for halogenated anesthetic vapors such as sevoflurane and desflurane [7, 8]

Total intravenous anesthesia (TIVA) is currently one of the preferred anesthetic methods, and it might be associated with the administration of alpha and/or beta blockers and halogenated or local anesthetics [9, 10].

In literature there is a scantiness of data about the use of light sedation with or without analgesia in pregnant patients who need to undergo surgical interventions [11, 12].

For such reason, it should be of paramount importance to avoid the use of inhaled anesthetics, which could induce fetal damages, especially during the first trimester of pregnancy. In addition, this kind of approach may prevent patient intubation, reducing possible traumatic events to the airways.

Reports concerning the use of analgosedation for head and neck surgery in pregnant women are extremely rare, and the only cases described in literature are about eye and dental surgery [13, 14].

## Case presentation

We present the case of a Caucasian 36-year-old woman during her 21st week of pregnancy, with a normal-sized fetus according to the gestational age on ultrasound exam and with no additional risk factors; she had no other pathologies and did not take any medications.

The patient referred to an nasal obstruction associated with rhinorrhea of the left nasal cavity starting 6 months earlier, with worsening of symptoms during the first trimester of pregnancy. She also reported episodes of sleep apnea and hyposmia.

The patient received a detailed otolaryngological examination, which allowed identification of a mass within the left nasal cavity.

The differential diagnosis of an intranasal mass includes benign causes, as well as malignant causes, such as squamous cell carcinoma (SCC), adenocarcinoma, nasal lymphoma, and others. So, it is important to carefully evaluate a patient with an intranasal mass, particularly if the mass is unilateral, which could be a malignancy sign, or there are other associated complaints, such as rapid growth [15].

**Fig. 1:**
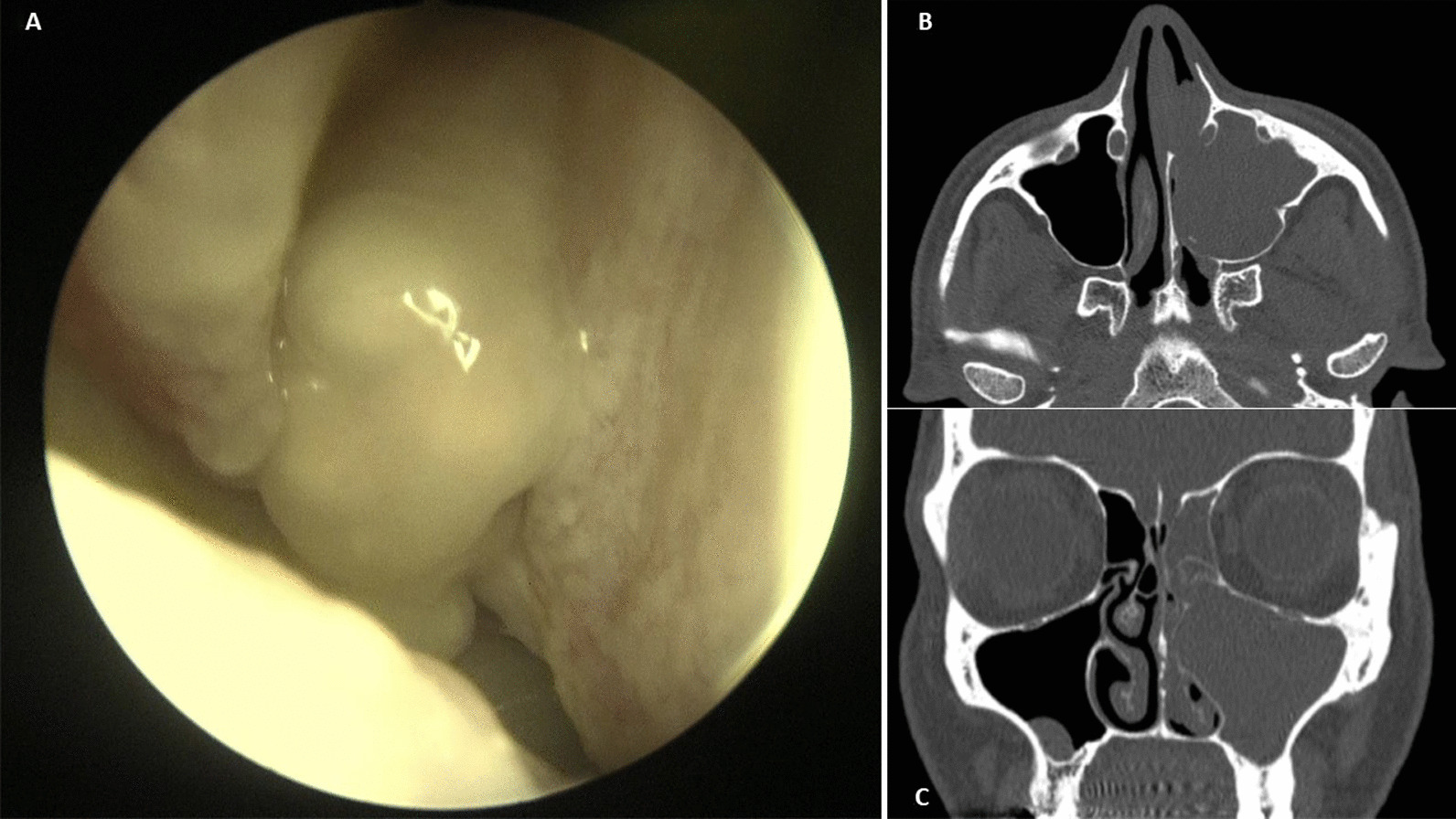
**a** Endoscopic appearance of a grayish polypoid mass lesion with a multinodular surface occupying the entire left nasal fossa, **b** Head CT scan: a axial view, **c** coronal view. Evidence of a lesion, 24 mm × 28 mm × 26 mm in size, totally obliterating the left maxillary sinus, resulting in obstruction of the anterior osteomeatal unit and ethmoidal sinusitis

**Fig. 2:**
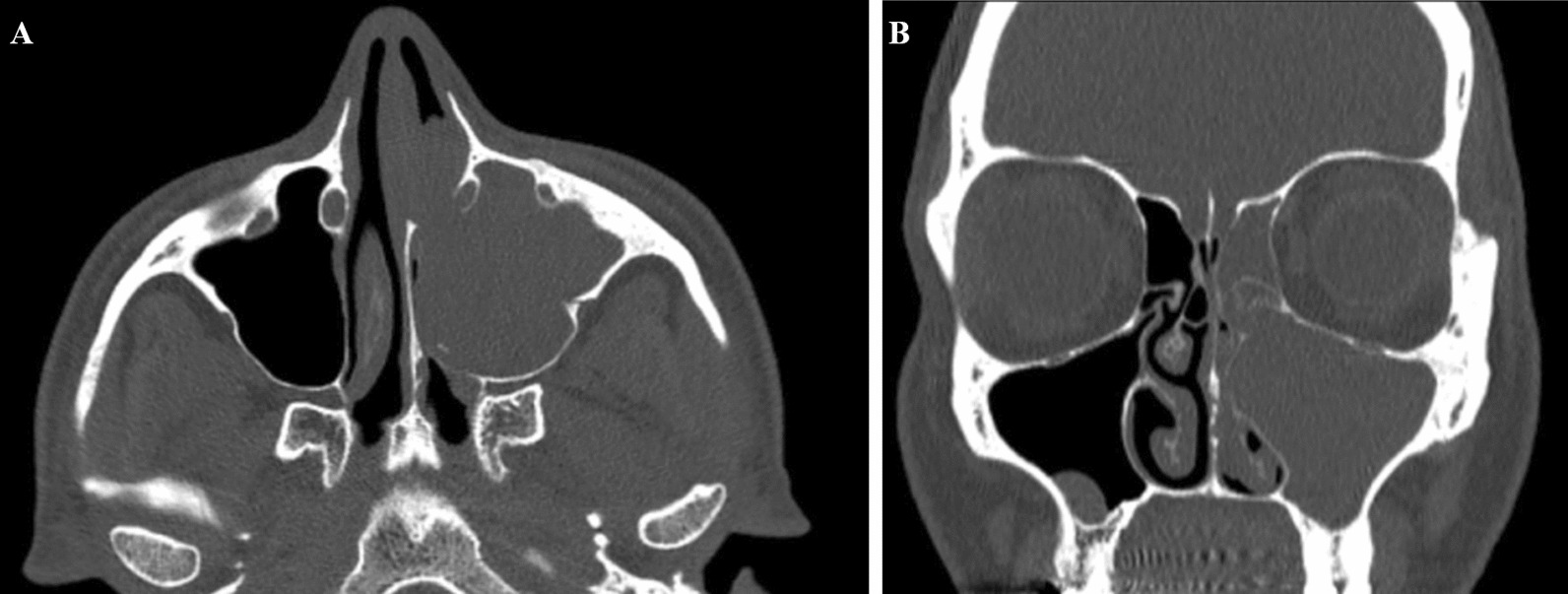
Head CT scan: **a** axial view, **b** coronal view. Evidence of a lesion, 24 mm × 28 mm × 26 mm in size, totally obliterating the left maxillary sinus, resulting in obstruction of the anterior osteomeatal unit and ethmoidal sinusitis

The subsequent nasal endoscopy confirmed a grayish polypoid mass lesion with a multinodular surface occupying the entire left nasal fossa (Fig. 1). Further imaging studies included a head computed tomorgraphy (CT) scan and a magnetic resonance imaging (MRI) of the paranasal sinuses that showed a 24 mm × 28 mm × 26 mm neoformation, hyperintense in T1 and hypointense in T2, with a convoluted cerebriform pattern. The lesion totally obliterated the left maxillary sinus, resulting in obstruction of the anterior osteomeatal unit and ethmoidal sinusitis (Fig. 2). The next healthcare step would have been the biopsy, but an in-office biopsy of a highly vascularized mass and area could lead to dangerous bleeding.

The adequate surgical treatment in such conditions should be a functional endoscopic sinus surgery (FESS) with excision of the nasal lesion associated with a left antrostomy and anteroposterior ethmoidectomy.

Therefore, the patient was evaluated in a multidisciplinary setting and received a full gynecological examination and fetal monitoring, which did not reveal any anomalies.

To have as minimally invasive of an approach toward the fetus as possible, the anesthetist proposed an analgosedation with spontaneous breathing.

The patient consented to the surgical and anesthetic procedure and signed a specific informed consent. During the preoperative anesthetic visit, the risk of airway management was carefully assessed, in relation to both the type of surgery and the fact that she was pregnant.

She was transferred to the postanesthesia care unit (PACU), where it was reconfirmed that she had no allergies and that she had followed an adequate fast.

A venous access was placed with a 18G cannula needle into the back of her right hand.

The patient was carried on a stretcher to the operating room and placed in a supine position on the surgical bed, with a wedge under the right side to facilitate venous return.

Her vital parameters were monitored for the entire duration of the intervention. Electrocardiogram (ECG) monitoring using lead II showed sinus rhythm, and her heart rate (HR) was 80 beats per minute (bpm). Her noninvasive blood pressure (NIBP) measured normal blood pressure (BP; 120/80 mmHg). SpO_2_ was equal to 98–99% at fraction of inspired oxygen of 21%, and respiratory rate (RR) was equal to 16 acts/minute.

In the operating room, the kit for the management of difficult intubation was set up with a videolaryngoscope and fiberoptic bronchoscope, as well as drugs and aids for general anesthesia. The patient was premedicated with 0.5 mg of intravenous midazolam. After 5 minutes, to start analgosedation in association with surgical local anesthesia (lidocaine 10 mg/ml with total volume 5 ml), the target-controlled infusion was set with remifentanil at a 50 mcg/ml concentration and target camptothecin (Cpt) of 1.5 ng/ml. This dosage was maintained for the entire duration of the surgery and allowed a Richmond agitation–sedation scale (RASS) of −1 and a visual analogue scale (VAS) 0.

The duration of the operation was 90 minutes, and every 15 minutes a bolus of 0.5 mg of midazolam was administered to titrate the concentration of the drug to maintain the desired level of sedation at the minimum necessary dosage. The continuous monitoring detected the following parameters before the operation started: BP 120/80 mmHg, SpO_2_ 99%, RR 14 breaths per minute, and HR 80 bpm. All parameters remained stable during the entire time of surgery and settled at: BP 110/60 mmHg, HR 70 bpm, RR 11 bpm, and SpO_2_ 97%. The patient was kept in spontaneous breathing, ensuring valid airway protective reflexes, such as cough, expiration reflex, and swallowing reflex, and making her promptly alert during the entire time of the surgical procedure.

After the surgical intervention, the patient was monitored in the PACU. Her hemodynamics were stable (within normal limits), and the VAS was 0. There was no need for any type of additional analgesic; also the patient had neither nausea nor vomiting.

The gynecological examination and fetal monitoring performed after the surgical procedure did not show any kind of abnormalities.

The postoperative hospitalization lasted 24 hours, and the patient did not present any complications.

Afterwards the patient was regularly assessed with clinical monitoring in the otolaryngology clinic. The final histological examination of the lesion turned out to be an inverted papilloma.

Postoperative follow-ups were performed by performing endoscopies at 1, 3, and 6 months and 1 year, without signs of disease recurrence.

Further confirmation was provided by MRI control 1 year after surgery.

## Discussion and conclusion

Pregnant women undergo different physiological adaptations, due not only to hormonal factors but also to mechanical and metabolic elements resulting from the increase in size of the uterus and the growing fetal demands [16].

The respiratory system in particular faces major changes, including a 20% increase in oxygen consumption and a decrease in lung capacity. Such adaptations are responsible for the increased risk of hypoxemia related to the induction of general anesthesia [17].

Another possible change reported in literature is edema of the oropharyngolaryngeal tissues; such edema results in a reduced glottic opening and difficulty in ventilation and tracheal intubation, starting from the second trimester of pregnancy [18, 19].

It is well known that, when a pregnant woman receives drugs, some cross the placenta, reaching fetal circulation through various mechanisms and possibly interacting directly with the fetus.

For instance, there are several studies performed in rats that have highlighted embryonic brain development after exposure to sevoflurane. Sevoflurane exposure at concentrations from 2% to 4% for 2 hours induces excessive levels of autophagy in the fetal brain through the activation of PTEN/Akt/mTOR pathway [5].

Usually, fetal brain cells are characterized by a continuous turnover: those cells which have accumulated damaged proteins and organelles undergo apoptosis through the PTEN transduction pathway. If the fetus is exposed to increased concentrations of sevoflurane, the apoptotic pathway undergoes dysregulation due to the excessive activation of PTEN. The physiological cell proliferation is compromised with consequent neurotoxicity. Furthermore, propofol and desflurane can also induce neuronal apoptosis [6].

On the contrary, administering opioids during pregnancy, especially remifentanil, is safer mainly due to its peculiar pharmacokinetics; in fact, it is quickly metabolized, avoiding interferences with the physiological changes of pregnancy and the fetus, as well [7, 8].

In literature, the reported incidence of head and neck neoplasms during pregnancy is rare (0.1% of all pregnancies), and oral cavity neoplasms account for most cases [20].

Unfortunately, sometimes neoplastic lesions might be confused with rhinitis, a common disorder in pregnant women and frequently associated with a preexisting asthmatic picture [21].

In this regard, typically Burkitt lymphoma and dematiaceous fungal infections are more frequent, especially among women who are immunocompromised or affected by aplastic anemia [22, 23].

FESS represents the most common surgical procedure for treatment of nasal sinus diseases not responsive to drug therapy [24].

Total intravenous anesthesia (TIVA) is currently one of the anesthetic methods of choice, frequently associated with the administration of alpha and/or beta blockers and halogenated and local anesthetics [9, 10].

The most common intraoperative occurrence during FESS, especially in the ethmoid and sphenoid sinus surgery, is represented by bleeding; therefore, maintaining normal blood pressure is of paramount importance. In fact, even minimal bleeding could compromise the visualization of the operating field, thus increasing the risk of iatrogenic lesions [25, 26].

In literature, there are very few studies concerning the use of superficial sedation with or without analgesia in pregnant patients who need to undergo surgery for nonobstetric indications [11, 12].

To the very best of our knowledge, there are no available data about analgosedation and ears, nose, and throat (ENT) surgery in pregnant women.

Interestingly, the use of superficial sedation maintains physiological homeostasis of pregnant woman with improvements in both perfusion and oxygenation. In fact, general anesthesia can affect uterine blood flow both for alterations in perfusion pressure and for variations in vascular resistance. Furthermore, maintaining spontaneous ventilation can prevent oxygenation defects that could lead to an increased risk of fetal asphyxia.

A key point, which needs to be clearly defined, is that the correct choice of the patient for patient’s compliance is essential; for example, the pregnant woman must be able to precisely follow the surgeons and anesthetists’ instructions during the operation.

For such reason, it is crucial to explain in detail the anesthetic techniques that are going to be used, specifically focusing on risks and benefits, thus motivating the patient.

## Data Availability

Data used during the current study is available from the corresponding author on reasonable request.

## Abbreviations

FESS: Functional endoscopic sinus surgery
TIVA: Total intravenous anesthesia
CT: Computed tomography
MRI: Magnetic resonance imaging
PACU: Postanesthesia care unit
ECG: Electrocardiography
HR: Heart rate
bpm: Beats per minute
NIBP: Noninvasive blood pressure
BP: Blood pressure
SpO_2_: Peripheral oxygen saturation
